# Pediatric meningioma and seizures: A scoping review

**DOI:** 10.1007/s00381-025-06889-z

**Published:** 2025-07-10

**Authors:** Emma Ye, Ashwin Gupta, Alexander T. Lyons, Shilpa B. Reddy, Devang J. Pastakia, Michael C. Dewan

**Affiliations:** 1 Vanderbilt University School of Medicine, Nashville, TN USA; 2 Department of Pediatric Neurology, Vanderbilt University Medical Center, Nashville, TN USA; 3 Department of Pediatric Hematology/Oncology, Vanderbilt University Medical Center, Nashville, TN USA; 4 Department of Neurological Surgery, Division of Pediatric Neurological Surgery, Vanderbilt University Medical Center, Nashville, TN USA

**Keywords:** Pediatric meningioma, Seizures, Surgical resection, Molecular features, Anti-seizure medication

## Abstract

**Objective:**

Pediatric meningiomas (PM) are rare tumors with unique clinical characteristics, including their association with seizures. This review aims to explore the available evidence regarding the nature of this association, the types of evidence available, and the gaps in our understanding.

**Methods:**

We synthesized evidence from systemic literature search. We identified key clinical concepts including the frequency of seizures as a presenting feature, seizure outcomes following surgical treatment, the relationship between extent of resection and seizure freedom, the impact of histologic features or grade on seizure freedom, and the role of post-operative anti-seizure medications (ASMs). Two proportion *z*-tests were performed with a *p* < 0.05 significance threshold.

**Results:**

In our search, we found 824 pediatric meningioma cases, with 293 (35.6%) presenting with seizure(s) at the time of meningioma diagnosis. Varying information regarding tumor grade, recurrence, seizure outcomes, and use of ASM was available in all 293 patients. One hundred-thirteen cases had a listed meningioma grade, with 80 (70.8%) classified as grade I, 19 (16.8%) as grade II, and 14 (12.4%) as grade III. 12 of the 113 patients with graded tumors (10.6%) received post-operative ASMs. Post-operative seizure status was reported in 76 of these patients and 50 (65.8%) achieved seizure freedom. Twenty-one underwent subtotal resection and only 2 of 7 (28.6%) were known to have had seizure freedom.

**Conclusions:**

Seizures are a recognized clinical feature in pediatric meningioma, occurring in approximately 36% of patients. They may present at diagnosis or emerge as a postoperative complication, with implications for long-term neurological outcomes. Surgical resection remains the standard of care for pediatric meningioma, and a greater extent of resection improves seizure outcomes. The role of post-resection ASMs and the relationship between seizures and molecular characteristics of pediatric meningioma warrants further investigation.

## Introduction

**Fig. 1:**
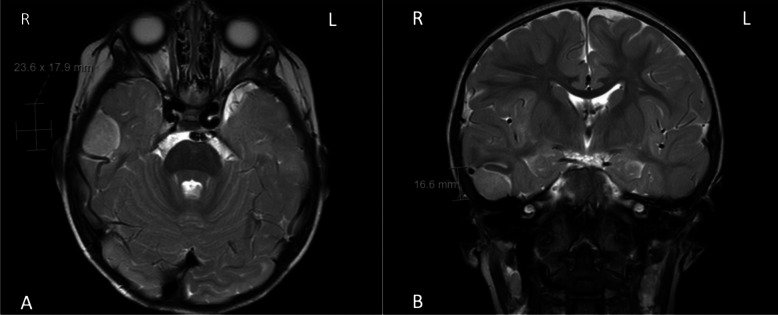
Axial (**A**) and coronal (**B**) T2-weighed MRI images demonstrate a rounded intradural, extra-axial lesion in the inferolateral temporal lobe with associated local mass effect and close association with the vein of Labbe. On contrasted images (not shown), the lesion homogeneously enhances

**Fig. 2:**
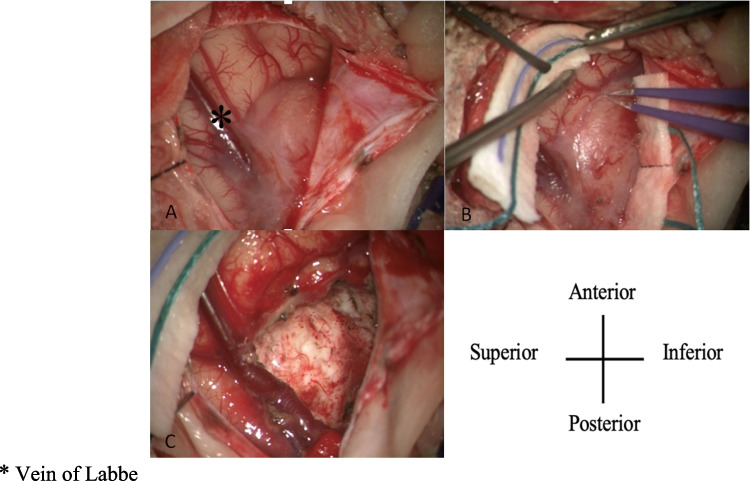
Intraoperative images of the right temporal exposure as viewed through the operating microscope. Screen top is patient anterior, screen left is patient superior. The fleshy extra-axial mass demonstrates growth around the vein of Labbe (**A**, **B**) as it becomes enveloped traveling inferior posteriorly. Following resection there is no evidence of microscopic residual disease, and the skeletonized vein of Labbe remains patent (**C**)

Pediatric meningiomas are rare central nervous system tumors, representing 1–2% of primary pediatric intracranial tumors [1, 2]. For adults, meningiomas represent a much larger share of primary brain tumors, at approximately 35% [2, 3]. Unlike adults, where a female predominance is well established, pediatric meningiomas show a more complex sex distribution that varies with pubertal status. Specifically, male predominance is observed in prepubertal children, while female predominance emerges post-puberty [2, 4, 5]. The age at presentation is typically between 10 and 14 years, although cases can occur throughout childhood [4]. Pediatric meningiomas are often more aggressive, have higher World Health Organization (WHO) grading, have greater association with genetic conditions such as neurofibromatosis type II (NF2), and have a higher risk of recurrence compared to adult meningiomas [2]. Prior cranial irradiation is also a well-recognized risk factor for the development of meningiomas in children. Radiation-induced meningiomas (RIMs) often present years after treatment for leukemia or other primary CNS malignancies and may differ biologically and clinically from sporadic cases [6]. Pre-operative MRI images and intra-operative images of a pediatric meningioma can be seen in Figs. 1 and 2, respectively. While pediatric meningiomas are rare, their diverse characteristics and potential for altering the developing brain present unique challenges. Presentations vary widely, including seizures, visual disturbances, motor deficits, cranial nerve palsies, or symptoms of increased intracranial pressure such as headache, nausea, vomiting, and papilledema [7]. Among the varied clinical symptoms, acute seizure activity is one of the most notable signs, and often presents challenges in management and long-term outcomes. Seizures are more commonly associated with pediatric meningiomas as compared to adults, as a meta-analysis found the preoperative seizure rate in pediatric meningiomas to be 42% as compared to 24% in adults [8]. Currently, the mechanism and pathophysiology of meningioma-associated seizures are not fully elucidated [9]. There remain gaps in our understanding of the frequency of seizure activity in pediatric meningiomas, their association with the WHO tumor grade and molecular features, the role between extent of resection (EOR) and seizure freedom, and the role of ASMs. This scoping review aims to synthesize existing evidence, identify gaps in knowledge, and outline key clinical concepts related to pediatric meningiomas and seizures.

## Methods

### Database search

A systematic review adhering to the PRISMA guidelines was conducted to explore the relationship between pediatric meningioma and seizures [10]. The study involved an extensive literature search of PubMed and Embase databases conducted in January 2025. There were no restrictions on publication date, study location, or design. The search strategy employed broad vocabulary terms for pediatrics, meningioma and seizure utilizing the PICO model [11]. The references contained in retrieved articles were subsequently examined to identify additional relevant studies. A complete list of search terms is included in supplementary Table 1.

### Screening process

We utilized the automated de-duplication setting in Covidence to remove duplicates. Every article was then reviewed primarily by title and abstract, with a full-text review performed by two independent reviewers (EY and AG) to ensure consistency in selection criteria and a comprehensive review followed by adjudication from the senior author (MCD). The inclusion criteria were defined as: publication in a peer-reviewed journal, presentation of primary pediatric meningioma and seizure data, and inclusion of a proportion of pediatric meningioma patients with seizures or a manner that allowed us to distinguish pediatric data. The search included case reports, case series, retrospective cohort analyses, literature reviews, and other relevant publications. There were no restrictions on the publication date or from where the study was conducted. The pediatric age range used was 0 to 18 years. The exclusion criteria included studies that focused solely on adult meningioma data and those without seizure rates, or a method for calculating seizure incidence in pediatric meningioma cases. Any differences or discrepancies were reconciled through consultation with the senior author.

### Data extraction, data reporting, and quantitative analysis

The studies included for final review were examined and relevant information on bibliographical information, study type, percentage of patients with seizure, seizure outcome, tumor grade, histologic features, and seizure prophylactic data was extracted. Data were extracted on study type, level of detail (patient-specific vs. cohort-wide), sample size, geographic location, demographic information, seizure frequency as a presenting feature, outcomes following surgical treatment, WHO tumor grade, histologic features, molecular features and the role of anti-seizure medications (ASM). EOR was classified according to the Simpson grade scale with Grades I–III classified as gross total resection (GTR) and Grades IV–V as subtotal resection (STR) [12, 13]. Data were reported as proportions where appropriate. Data analysis was conducted in Microsoft Excel. Two proportion *z*-tests were conducted with *p* < 0.05 used as the threshold for significance.

## Results

**Fig. 3:**
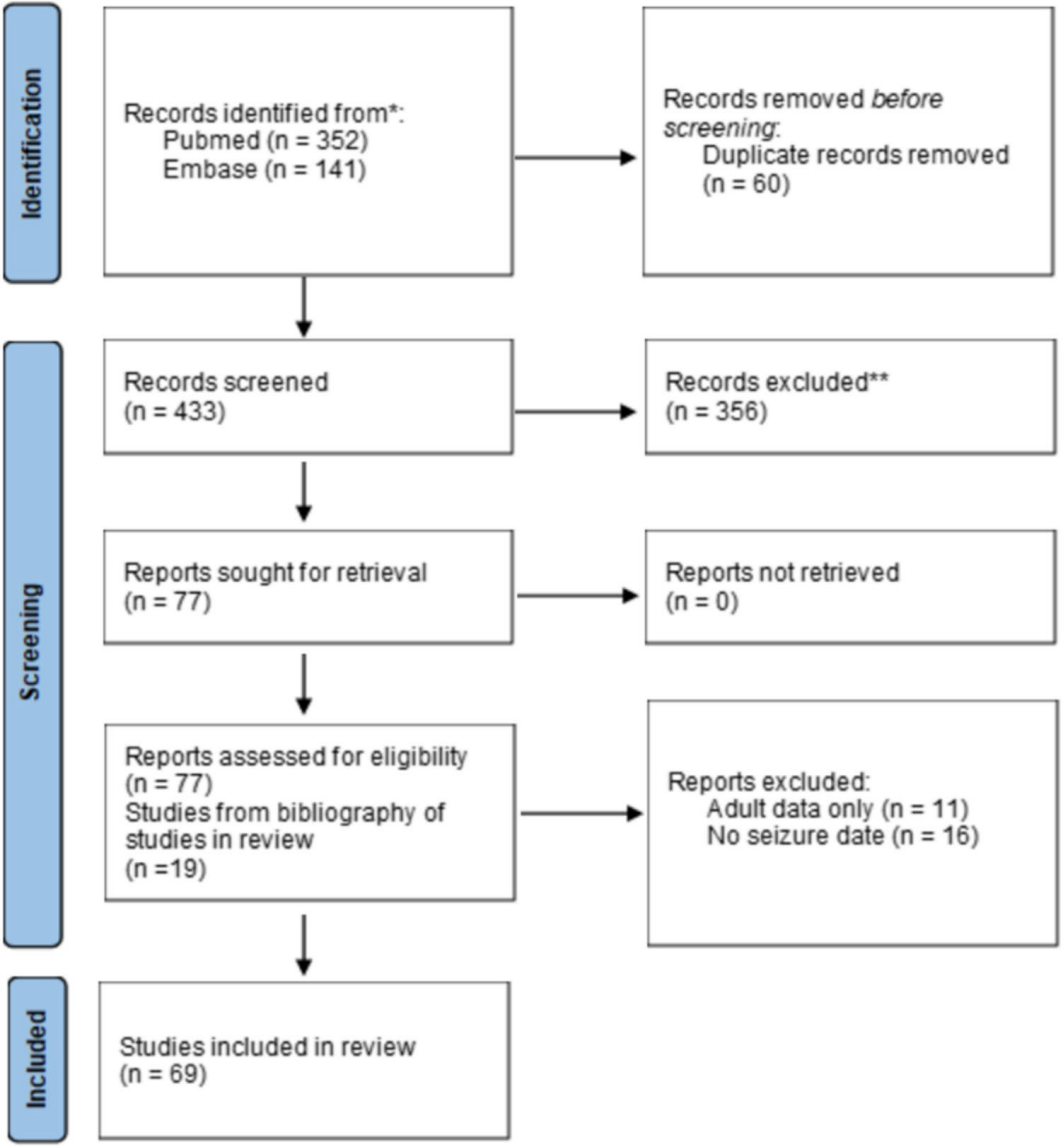
Prisma (Preferred reporting items for systematic reviews and meta-analysis) flow diagram for the literature search

The initial literature query yielded 493 studies: 352 from PubMed and 141 from Embase. 60 duplicate studies were automatically identified by Covidence and removed, leaving 433 studies remained. After a primary review of the abstracts, 77 studies remained, and 53 studies met the inclusion criteria for data extraction. 19 studies were identified through a bibliography review of these manuscripts and included as relevant literature. Among these 69 studies, 25 (36.2%) were retrospective cohort analyses, 32 (46.4%) were case reports, and 12 (17.4%%) were case series. The PRISMA flowchart is presented in Fig. 3. The risk of bias for each study was determined from the ROBINS-I tool (BMJ)[14].

### Demographics of pediatric meningiomas

Across all included studies, we identified 824 cases of pediatric meningiomas that met the inclusion criteria. Of these 824 cases, 293 (35.6%) presented with seizure at the time of diagnosis. Among these 293 patients, there was variable information reported regarding WHO tumor grade, tumor recurrence, resolution of seizures, histologic and molecular features, and long-term use of ASMs. 113 patients had a listed grade, with 80 (70.8%) classified as grade I, 19 (16.8%) as grade II, and 14 (12.4%) as grade III. Of these patients, 112 had a listed age and sex; 65 (58%) were male and 47 (42%) were female. The mean age was 9 years with a standard deviation of 5.3 years (median 9 years, interquartile range 5–13 years). There was information regarding NF2 status in 70 graded pediatric meningiomas, and 2 (2.9%) patients had seizures as initial presenting symptoms.

### Seizure at presentation in pediatric meningiomas

Our literature review found a cumulative seizure frequency of 35.6% at the presentation of pediatric meningioma across 69 studies. We identified 293 patients presenting with acute seizure activity out of 824 recorded pediatric meningioma cases.

### Extent of resection and seizure outcome

We identified 85 patients who underwent GTR, 31 of whom had seizure-related data after surgery. Of those 31, 25 (80.6%) achieved post-operative seizure freedom while 6 (19.4%) continued to have seizures after surgery. Among the 25 patients with seizure freedom, 15 (68.2%) had a WHO grade I tumor, 5 (22.7%) had a grade II tumor, and 2 (9.1%) had a grade III tumor; 3 patients had an unknown WHO tumor grade. Of the 6 cases without seizure freedom, 4 tumors were grade I (80.0%), 1 was grade III (20.0%), and 1 had an unknown WHO tumor grade. On the other hand, there were 21 cases of patients receiving STR, only 7 for whom seizure-related data was reported after surgery. Two (28.6%) patients achieved seizure freedom, in which one had a grade I tumor and the other had a grade III tumor. The remaining 5 (71.4%) did not report seizure freedom post-operatively; 3 (60.0%) were grade I and 2 (40.0%) were grade II. There was a significantly higher rate of seizure freedom following GTR compared to STR (25/31 and 2/7, respectively) (*P* = 0.0061).

### Histopathologic subtype and seizures

Limited data was available regarding the intersection between histologic features, seizure incidence and outcome. We found 102 cases of pediatric meningiomas with seizures as a presenting symptom that also listed histologic features. Our literature review revealed that meningothelial tumors were the most prevalent histologic subtype, with the highest incidence of seizures, comprising 29 cases 28.4%) of the overall cohort of 102 pediatric meningiomas presenting with seizures with a listed histologic subtype. Fibroblastic tumors were the second most common subtype, accounting for 18 cases (17.6%) of the total cohort. Transitional tumors followed, representing 15 cases (14.7%). Less frequently observed subtypes included atypical tumors (14 cases, 13.7%), malignant tumors (6cases, 5.9%), and papillary tumors (4 cases, 3.9%). Rare histologic subtypes included chordoid (3 cases, 2.9%), psammomatous (2 cases, 2.0%), angiomatous (2 cases, 2.0%), sclerosing (2 cases, 2.0%), microcystic (2 cases, 2.0%), anaplastic (2 cases, 2.0%), and rhabdoid tumors (2 cases, 2.0%). Clear cell tumors were the least common, accounting for 1 case (1.0%) in the overall cohort Table 1.

**Table 1:** Overview of Literatures Included in Study

Investigator	Study design	Study period	Country	Risk of bias	NF2 in graded seizure cases	Number of patients (seizures/total)	Initial grade of seizure cases	Histopathology of graded seizure cases	RiM of graded seizure cases	Molecular characteristics
Chan et al., 1984[15]	CS	1960–1981	Canada	Moderate	0/4	4/4	I (3), III (1)	Fibroblastic (2), Transitional (1), Malignant (1)	N/A	N/A
Ferrante et al., 1989[16]	RC	1952–1987	Italy	Moderate	N/A	6/19	I (5), III (1)	Meningothelial (3), Fibroblastic (2), Anaplastic (1)	N/A	N/A
Germano et al., 1994[17]	RC	1948–1990	Italy, USA	Moderate	N/A	6/23	N/A	N/A	N/A	N/A
Baumgartner et al., 1996[18]	RC	1964–1994	USA	Moderate	0/4	4/11	I (2), II (1), III (1)	Meningothelial (2), Atypical (1), Malignant (1)	N/A	N/A
Malluci et al., 1996[19]	RC	1957–1993	UK	Moderate	N/A	4/16	N/A	N/A	N/A	N/A
Kobata et al., 1998[20]	CR	1993	Japan	Moderate	0/1	1/1	II (1)	Chordoid (1)	N/A	N/A
Yoon et al., 1999[21]	CS	N/A	Korea	Moderate	0/3	3/5	I (2), III (1)	Meningothelial (1), Transitional (1), Malignant (1)	N/A	N/A
Amirjamshidi et al., 2000[22]	RC	N/A (15 years)	Iran	Moderate	0/6	6/24	I (6)	Meningothelial (3), Psammomatous (2), Fibroblastic (1)	N/A	N/A
Demirtas et al., 2000[23]	RC	1970–1995	Turkey	Moderate	N/A	7/16	N/A	N/A	N/A	N/A
Im et al., 2001[24]	CS	1981–1999	Korea	Moderate	1/5	5/11	I (5)	Meningothelial (2), Microcystic (1), Sclerosing (1), Transitional (1)	N/A	N/A
Lund-Johansen et al., 2001[25]	RC	1972–1999	Norway	Moderate	N/A	9/27	N/A	N/A	N/A	N/A
Symons et al., 2001[26]	CR	1998	Australia	Moderate	0/1	1/1	I (1)	Meningothelial (1)	N/A	N/A
Zwerdling et al., 2002[27]	RC	1974–1999	USA	Moderate	N/A	5/18	I (4), III (1)	Transitional (3), Fibroblastic (1), Malignant (1)	N/A	N/A
Rushing et al., 2005[28]	RC	1970–2004	USA	Moderate	N/A	29/87	N/A	N/A	N/A	N/A/
Tufan et al., 2005[29]	RC	1983–2003	Turkey	Moderate	0/1	1/11	III (1)	Malignant (1)	N/A	N/A
Mullassery et al., 2006[30]	CR	N/A	UK	Moderate	0/1	1/1	II (1)	Chordoid (1)	N/A	N/A
Avninder et al., 2007[31]	CR	N/A	India	Moderate	0/1	1/1	III (1)	Papillary (1)	N/A	N/A
Alexiou et al., 2008[32]	CS	1992–2007	Greece	Moderate	N/A	3/8	I (1), II (2)	Atypical (2), Meningothelial (1)	N/A	N/A
Arivazhagan et al., 2008[33]	RC	1990–2005	India	Moderate	N/A	11/33	N/A	N/A	N/A	N/A
Greene et al., 2008[34]	RC	1978–2004	USA	Moderate	N/A	6/20	N/A	N/A	N/A	N/A
Liu et al., 2008[35]	RC	1986–2005	China	Moderate	0/6	6/12	N/A	N/A	N/A	N/A
Maranhão-Filho et al., 2008[36]	RC	1997–2007	Brazil	Moderate	0/4	4/7	I (3), III (1)	Meningothelial (2), Fibroblastic (1), Papillary (1)	N/A	N/A
Teixidor et al., 2008[37]	RC	1981–2003	Spain	Moderate	N/A	6/10	I (5), III (1)	Meningothelial (3), Transitional (1), Angiomatous (1), Anaplastic (1)	N/A	N/A
Gao et al., 2009[38]	RC	1993–2008	China	Moderate	N/A	19/54	N/A	N/A	N/A	N/A
Li et al., 2009[39]	RC	2000–2007	China	Moderate	N/A	6/34	N/A	N/A	N/A	N/A
Mehta et al., 2009[40]	RC	1974–2005	India	Moderate	N/A	8/18	N/A	N/A	N/A	N/A
Menon et al., 2009[41]	RC	1982–2005	India	Moderate	N/A	29/38	N/A	N/A	N/A	N/A
Lakhdar et al., 2010[42]	RC	1983–2007	Morocco	Moderate	0/6	6/21	N/A	N/A	N/A	N/A
Morina et al., 2010[43]	CR	2008	Kosovo	Moderate	0/1	1/1	III (1)	Rhabdoid (1)	N/A	N/A
Chohan et al., 2011[44]	CR	N/A	USA	Moderate	1/1	1/1	II (1)	Atypical (1)	N/A	169 C > T truncating mutation
Jaiswal et al., 2011[45]	CS	1999–2009	India	Moderate	0/5	5/12	I (4), II (1)	Meningothelial (3), Transitional (1), Atypical (1)	N/A	N/A
Shimbo et al., 2011[46]	CR	N/A	Japan	Moderate	0/1	1/1	I (1)	Meningothelial (1)	N/A	N/A
Suematsu et al., 1974[47]	CR	N/A	Japan	Moderate	0/1	1/1	I (1)	Fibroblastic (1)	N/A	N/A
Morimoto et al., 1976[48]	CR	N/A	Japan	Moderate	0/1	1/1	III (1)	Malignant (1)	N/A	N/A
Legius et al., 1985[49]	CR	1985	Belgium	Moderate	0/1	1/1	I (1)	Fibroblastic (1)	N/A	N/A
Drake et al., 1985[50]	CS	1934–1985	Canada	Moderate	0/3	3/13	I (3)	Transitional (3)	N/A	N/A
Schroeder et al., 1987[51]	CR	1987	USA	Moderate	0/1	1/1	I (1)	Fibroblastic (1)	N/A	N/A
Kimura et al., 1987[52]	CR	1987	Japan	Moderate	0/1	1/1	I (1)	Fibroblastic (1)	N/A	N/A
Matsumoto et al., 1992[53]	CR	N/A	Japan	Moderate	0/1	1/1	I (1)	Transitional (1)	N/A	N/A
Kaneko et al., 1993[54]	CR	1993	Japan	Moderate	0/1	1/1	I (1)	Meningothelial (1)	N/A	N/A
Kohama et al., 1996[55]	CR	1995	Japan	Moderate	0/1	1/1	I (1)	Fibroblastic (1)	N/A	N/A
Karadereler et al., 2004[56]	CR	N/A	Turkey	Moderate	0/1	1/1	I (1)	Fibroblastic (1)	N/A	N/A
Zhang et al., 2006[57]	CR	2005	China	Moderate	0/1	1/1	II (1)	Atypical (1)	N/A	N/A
Jung et al., 2012[57]	CR	2010	Korea	Moderate	0/1	1/1	I (1)	Transitional (1)	N/A	N/A
Manwaring et al., 2012[58, 59]	CR	N/A	USA	Moderate	0/1	1/1	I (1)	Microcystic (1)	N/A	N/A
Santos et al., 2012[58]	RC	1994–2010	Brazil	Moderate	0/3	3/15	I (2), II (1)	Transitional (1), Atypical (1), Meningothelial (1)	2/3	N/A
Ravindranath et al., 2013[60]	RC	1988–2012	India	Moderate	N/A	11/31	N/A	N/A	N/A	N/A
Ahmed et al., 2015[61, 62]	CR	N/A	USA	Moderate	0/1	1/1	I (1)	Angiomatous (1)	N/A	Gain of function in GNAQ
Donovan et al., 2016[62]	CS	N/A	USA	Moderate	0/2	2/2	I (2)	Meningothelial (1), Fibroblastic (1)	N/A	N/A
Larrew et al., 2016 [63]	CR	N/A	USA	Moderate	0/1	1/1	I (1)	N/A	N/A	N/A
Silbergeld et al., 1988	CR	1988	USA	Moderate	0/1	1/1	I (1)	Meningothelial (1)	N/A	N/A
Cho et al., 1990 [64]	CR	N/A	Korea	Moderate	0/1	1/1	I (1)	Transitional (1)	N/A	N/A
Mitsuyama et al., 2000 [65]	CR	1999	Japan	Moderate	0/1	1/1	I (1)	Fibroblastic (1)	N/A	N/A
Kumar et al., 2009 [66]	CR	2008	India	Moderate	0/1	1/1	I (1)	N/A	N/A	N/A
Aras et al., 2013 [67]	CR	N/A	Turkey	Moderate	0/1	1/1	I (1)	Fibroblastic (1)	N/A	N/A
Fukushima et al., 2014 [68]	CR	2013	Japan	Moderate	0/1	1/1	I (1)	Sclerosing (1)	N/A	N/A
Kaplan et al., 2002 [69]	CR	2001	USA	Moderate	0/1	1/1	II (1)	Atypical (1)	N/A	N/A
Tan et al., 2018 [70]	CR	N/A	Singapore	Moderate	0/1	1/1	I (1)	Fibroblastic (1)	N/A	N/A
El Beltagy et al., 2019 [71]	RC	2007–2017	Egypt	Moderate	N/A	17/39	N/A	N/A	N/A	N/A
Amirjamshidi et al., 2019 [22, 72]	CS	1986–2018	Iran	Moderate	0/2	2/3	I (2)	Meningothelial (2)	N/A	N/A
Hong et al., 2019 [72]	CS	2002–2017	Japan	Moderate	0/2	2/5	II (2)	Atypical (2)	N/A	N/A
Thevandiran et al., 2020 [73]	CS	1998–2018	Singapore	Moderate	0/4	4/10	I (2), II (2)	Fibroblastic (1), Chordoid (1), Atypical (1), N/A (1)	N/A	N/A
Liu et al., 2021[74]	RC	2003–2017	China	Moderate	N/A	7/40	N/A	N/A	N/A	N/A
Santana-Gonzalez et al., 2021[75]	RC	1992–2019	Mexico	Moderate	N/A	8/44	I (8)	N/A	N/A	N/A
Takata et al., 2021[76]	CR	N/A	Japan	Moderate	0/1	1/1	II (1)	Atypical (1)	N/A	N/A
Opoku et al., 2022[1]	CS	2012–2021	China	Moderate	0/1	1/10	III (1)	Papillary (1)	N/A	N/A
Chong et al., 2019[77]	CR	N/A	Ecuador	Moderate	0/1	1/1	I (1)	Meningothelial (1)	N/A	NA
Hui et al., 2015[78]	CS	2001–2011	India	Moderate	N/A	4/15	II (2), III (2)	Rhabdoid (1), Papillary (1), Clear cell (1), Atypical (1)	N/A	NA
Hafiz et al., 2024[79]	CR	N/A	Saudi Arabia	Moderate	0/1	1/1	II (2)	Atypical (1)	N/A	NA

*CR*, Case Report; *CS*, Case Series; *N/A*, Not Available; *RC*, Retrospective cohort; *RiM*, Radiation induced meningioma

Risk of Bias from ROBINS-I: https://doi.org/10.1136/bmj.i4919; Grade: Grading of recommendations, assessment, development and evaluations

We had 11 post-operative seizure recurrent cases in total, with 10 having information about histology and WHO grades. Table 2 outlines the histology frequency among all pediatric meningioma patients with seizures along with the recurrent seizure cases. A significant difference was observed in the distribution of histological subtypes between seizure-recurrent and non-recurrent cases of pediatric meningiomas. Meningothelial cases were significantly less frequent in seizure-recurrent patients, with 29 cases (28.4%) in the overall cohort with acute seizure activity as an initial presenting symptom and none (0%) in the seizure-recurrent group (*P* = 0.049). Fibroblastic cases were more frequent in seizure-recurrent patients, with 18 cases (17.6%) in the overall cohort and 5 cases (50.0%) in the seizure-recurrent group (*P* = 0.016). Transitional cases were observed in 15 patients (14.7%) overall and in 3 patients (30.0%) in the seizure-recurrent group, but the difference was not statistically significant (*P* = 0.21). Atypical meningiomas accounted for 14 cases (13.7%) overall and none (0%) in the seizure-recurrent group (*P* = 0.21). Malignant meningiomas were present in 6 cases (6.3%) overall and none (0%) in the seizure-recurrent group (*P* = 0.43). There were no significant differences between the proportion of tumor grades in the overall cohort and seizure recurrence.

**Table 2:** Pediatric Meningioma Seizure Data. *N* is the number of non-missing values. Numbers after frequencies are proportions. Tests used: ^1^Two proportion *z* test

Histology	Overall pediatric meningiomas with seizures and grades (N = 102)	Seizure recurrent patients with histology and grades (N = 10)	*P*-value
Meningothelial	29 (28.4%)	0 (0%)	***P* = 0.049**^**1**^
Fibroblastic	18 (17.6%)	5 (50.0%)	***P* = 0.016**^**1**^
Transitional	15 (14.7%)	3 (30.0%)	*P* = 0.21^1^
Atypical	14 (13.7%)	0 (0%)	*P* = 0.21^1^
Malignant	6 (5.9%)	0 (0%)	*P* = 0.43^1^
Papillary	4 (3.9%)	0 (0%)	*P* = 0.52^1^
Chordoid	3 (2.9%)	1 (10.0%)	*P* = 0.25^1^
Psammomatous	2 (2.0%)	0 (0%)	*P* = 0.65^1^
Angiomatous	2 (2.0%)	0 (0%)	*P* = 0.65^1^
Sclerosing	2 (2.0%)	0 (0%)	*P* = 0.65^1^
Microcystic	2 (2.0%)	0 (0%)	*P* = 0.65^1^
Anaplastic	2 (2.0%)	0 (0%)	*P* = 0.65^1^
Rhabdoid	2 (2.0%)	1 (10.0%)	*P* = 0.13^1^
Clear Cell	1 (1.0%)	0 (0%)	*P* = 0.76^1^

### Molecular features and seizure

Our literature review identified 2 out of 70 patients diagnosed with NF2 who had acute seizure activity as an initial presenting symptom of their meningioma. Notably, one NF2 case involved a spontaneous 169– > T truncating mutation with no family history of NF2. Additionally, we observed a reported pediatric angiomatous meningioma that revealed GNAQ gain of function mutation associated with Sturge-Weber syndrome. Specific molecular alterations identified as risk factors for seizures in PM were not found in this review. Most of the studies included in this review were published prior to the widespread use of next genome sequencing (NGS), and thus did not report comprehensive molecular profiling.

### Radiation-induced meningioma and seizure

We identified a single study exploring RiM in the pediatric population [58]. Among the 15 cases of meningiomas, 6 were associated with radiation-induced meningiomas. Of the 3 cases presenting with seizure activity as the initial symptom, 2 involved radiation-induced meningiomas.

### Anti-seizure medications (ASMs) for pediatric meningioma

There was limited reporting on the use of ASM for PM. Among 21 pediatric meningioma cases with documented ASM use, 3 patients (14.3%) were given ASM before surgery, with no post-operative seizure activity recurrence. 18 (85.7%) of the 21 patients received post-operative ASMs, with 3 (16.7%) patients experiencing persistent drug-resistant epilepsy while 15 patients (83.3%) enjoyed seizure freedom. Of these 18 patients, 6 (33.3%) had WHO grade I tumors, 4 (22.2%) had Grade II tumors, 2 (11.1%) had grade III tumors, and 6 (33.3%) had unknown grades. There were no significant differences in seizure outcomes based on tumor grade.

Notably, 9 (75%) of the 12 patients with information on extent of resection underwent GTR, while 3 (25%) had STR. Of the 8 GTR cases, 100% achieved seizure freedom on post-operative ASMs. Notably, 2 patients who initially received post-operative ASM therapy were eventually weaned off their medications. Among the 3 STR cases, 2 patients achieved seizure freedom with ASMs, while 1 patient experienced persistent drug-resistant epilepsy despite ASM therapy and underwent a second surgery for tumor recurrence. This patient continued to have occasional seizures following the second surgery.

## Discussion

In this review, we have collated the most recent literature on the intersection of pediatric meningioma and acute seizure activity. Available evidence suggests that seizures are a recognized presenting symptom of pediatric meningiomas, with reported frequencies ranging from 30 to 50% [7–9]. Our frequency of 36% coincides with this previously understood data. However, the exact incidence may vary depending on factors such as tumor location, histological subtype, molecular features and tumor grade. Our analysis demonstrates that neither tumor histologic subtype nor grade shows a clear association with seizure occurrence or the need for ASM prophylaxis. In contrast, GTR, as defined by Simpson grades I–III, appears to correlate with higher rates of postoperative seizure freedom. However, this observation is likely influenced by a complex interplay of factors, including tumor location, surgical approach, and individual patient characteristics, which are not readily apparent in this method of study. These findings provide clinically relevant insights into seizure burden in pediatric meningioma and underscore the need for enhanced risk stratification and postoperative management strategies.

The EOR remains one of the most consistently discussed variables. As mentioned above, using the Simpson grading scale, we classified Grades I–III as gross total resection (GTR) and Grades IV–V as subtotal resection (STR) [13]. Consistent with prior literature [9], we observed that patients undergoing GTR had higher rates of postoperative seizure freedom. However, the optimal extent of resection for maximizing seizure freedom while minimizing neurological deficits remains to be elucidated and is likely to vary by patient. Our literature review shows several notable findings in the comparison between the GTR and STR groups. The GTR subset had higher rates of post-operative seizure freedom and lower rates of death. While this may be attributable to operative technique, the role of GTR candidacy in operative selection cannot be overlooked, as patients with complex tumors in critical regions may not be eligible. To account for this, we aimed to examine factors responsible for differences in outcomes between the two surgical interventions such as tumor size, location, grade, and more. It is important to note that tumor location, which also plays a key role in seizure risk, was not consistently reported across the included studies. As such, location could not be incorporated into this analysis, limiting our ability to fully evaluate the interaction between tumor site, extent of resection, and seizure outcomes. Regardless, in addition to promoting seizure freedom, current literature shows that pediatric patients who initially undergo GTR of meningioma have significantly better progression-free and overall survival than those with upfront STR [13].

Compared to adults, pediatric meningiomas are more frequently higher grade and more commonly present with seizures [9, 15]. While previous studies in adult populations have reported a postoperative seizure freedom rate of approximately 69%, there is a notable lack of literature on seizure outcomes following resection of pediatric meningiomas [8]. Our literature review reveals that 27 (35.5%) out of 76 patients with reported post-operative seizure-related data continued to experience epilepsy post-operatively. It is likely that those with higher grade tumors experience epilepsy at a greater rate due to an invasion of surrounding tissues. Epilepsies were controlled in 12 patients with ASM, while 3 patients continued to experience seizures despite ASM treatment. However, specific details regarding ASM regimens were not consistently reported. Mattey et al. reports a case of postoperative seizure in a patient who did not present with seizures as an initial symptom [80].

NF2 mutations have been well-documented in pediatric meningioma, with reported frequencies ranging from 8% in larger metanalysis to 50% in specialized hubs for concentrated pediatric NF2 care [14, 80, 81]. In this review, NF2 accounted for a small proportion of reported patients both with seizure presentation and seizure frequency. Our literature review found that only 2 (2.9%) out of 70 patients diagnosed with NF2 had acute seizure activity as initial presenting symptoms of their meningiomas. Regrettably, many studies that show a link between PM and NF2 do not provide information on seizure activity in relation to NF2. We noted a case of a pediatric NF2 patient with three separate meningiomas of unique grades, each in disparate locations, indicating the complex interplay between various molecular and genetic features [61]. No cases of NF1 with seizure as the initial presenting symptom were documented in our review. Further data reporting with increasing granularity is needed to elucidate a potential association between NF2, NF1 and seizure activity or tumor grade in PM. In addition to those associated with syndromes, pediatric meningiomas exhibit unique pathological and molecular features. Our literature review identified only 2 reported cases of PM with seizure that have detailed molecular features. The first case involved a spontaneous 169C– > T truncating mutation, which was found to be associated with NF2, although there was no family history of the condition[44]. The second case was a reported pediatric angiomatous meningioma in a patient with Sturge-Weber syndrome revealing GNAQ gain of function mutation [61]. Recent studies have begun to link somatic mutations with seizure risk. Over 80% of sporadic meningiomas fall into one of seven molecular subgroups, including NF2, TRAF7, SMARCB1, KLF4, and mutations in Hedgehog and PI3K pathways [2, 51, 81]. Gupte et al. found that NF2 mutations were associated with preoperative seizures, primarily through indirect effects mediated by atypical histology and peritumoral brain edema, rather than the mutation alone [12]. Although data on postoperative seizures remain limited, hedgehog pathway mutant meningiomas—typically located along the olfactory groove—may be linked to increased postoperative seizure risk due to associated bilateral frontal edema [42, 51].

Radiation-induced meningiomas are a rare side effect of cranial radiation often arising over a decade after treatment [6]. Despite their generally low prevalence, meningiomas are the most common secondary brain tumor following cranial radiation [82]. Additional effort was expended to identify the relation between radiation-induced meningiomas and seizures as a presenting symptom. The work by Santos et al. served as the only article describing both RiM and seizures with a table showing two patients diagnosed with RiM, which had age ranges from 13 to 18 [58]. Given the paucity of data, the association between seizures and RiM cannot be firmly established. The relatively long time horizon between radiation exposure and the development of meningioma (and seizures) likely contributed to the lower reported seizure rate in this review which excludes patients over 18 years of age.

Despite this work, further research could be performed in several identified areas of improvement. These include the direct relationship between EOR and seizure freedom, the impact of histologic grade and molecular alterations on seizure outcomes, and the role and efficacy of ASMs in pediatric meningioma. Several limitations to this study deserve mention. As with any literature review, the data extracted and analyzed is limited to that which was reported within source manuscripts. As a result, several of our subgroups, such as those with STR, were small in size. Aggregate data limited the number of comparison variables available to explore for comparison. In some cases, study subjects were excluded if their age or seizure status could not distinguish from those within a larger cohort. Individual studies variably defined the parameters of EOR, including the use or non-use of the Simpson scale. Notably, most of the studies included in this review predate the widespread adoption of whole next genome sequencing (NGS), limiting the ability to comprehensively characterize the molecular landscape of pediatric meningiomas. Additionally, seizure-freedom follow-up duration was heterogenous across studies, ranging from 3 months to 12 years. Lastly, there are no standardized guidelines for the use of ASMs in pediatric meningioma, thus comparing seizure outcomes between some cohorts is not possible. The limited data available often lacked granularity, precluding us from examining differences in medication regimens and their subsequent effects on seizure control. Beyond further investigation of seizure incidence at presentation, future studies should explore postoperative seizure management strategies, risk factors, and outcomes to improve patient care and prognosis. Large multicenter studies or prospective pragmatic trials may help define the efficacy and safety of ASMs in pediatric patients undergoing surgical resection for meningiomas.

## Conclusion

Seizures are a recognized clinical feature in pediatric meningioma, influencing disease presentation, treatment strategies, and long-term neurologic outcomes. This scoping review synthesizes current literature on the intersection between pediatric meningioma and seizure activity, highlighting areas of consistency and ongoing uncertainty. While our findings suggest that complete surgical resection may be associated with improved seizure freedom, they are limited by the absence of multivariate analysis accounting for tumor location, grade, histologic subtype, molecular alterations, and treatment differences. No consistent relationship was found between seizure outcomes and histologic subtype or tumor grade. The biological mechanisms underlying seizure development in pediatric meningioma remain poorly understood, and molecular profiling data are notably sparse. Future research should focus on elucidating molecular alterations that may predispose to seizures, identifying predictors of postoperative seizure persistence, and clarifying the role of prophylactic antiseizure medications in this population. Addressing these gaps is essential to improving seizure control, functional recovery, and overall quality of life in children with meningioma.
